# Supplementary material for: Bartlett-corrected tests for varying precision beta regressions with application to environmental biometrics
Source: PLoS One. 2021 Jun 28;16(6):e0253349. doi: 10.1371/journal.pone.0253349 (PMC8238208; doi:10.1371/journal.pone.0253349)
Supplement: Attachment — Submitted filename: reply-referees_plos-one_v1.pdf [file pone.0253349.s001.pdf]

REPLY TO THE EDITOR AND TO THE REFEREES

The manuscript entitled "Bartlett-corrected tests for varying precision beta regressions with application to environmental biometrics" was submitted to *PLOS ONE* (Manuscript Number: PONE-D-21-03109). The Academic Editor's decision, which was based on considerations made by two referees, was: "The manuscript has been assessed by two reviewers, both of whom suggest several points of improvement. We invite you to submit a revised version of the manuscript that addresses the points raised during the review process. Please submit your revised manuscript by May 24 2021 11:59PM."

AUTHORS' REPLY TO THE EDITOR: As detailed below, we took all comments and suggestions made by the two referees into account when revising our manuscript. The changes we made to the text are in blue typeface. Thank you for considering the revised version of our paper for publication.

REFEREE 1

REFEREE: The work is rigorous but omits the detailed derivation of the correction terms, which, in my opinion, should be made available as a supplementary file. In addition to that I have the following comments:

AUTHORS' REPLY: Thank you. We included the derivation of the Bartlett correction in the manuscript; see the Appendix.

REFEREE: 1. Authors have cited many classical old work in the field. It would be better if you cite some recent work and mention to see the reference in that article. e.g. your reference [22] can be used to cite the work of Bartlett (1937) and Lawley (1956). Both the work are more than 50 years old; are fundamental contribution in Statistics and can be found in any standard book of small area estimation. Similarly, for other old work.

AUTHORS' REPLY: Thank for the suggestion. We removed several historic and very old references from the text. We only kept a citation to Lawley's work since it is very important for our analysis. We hope you will agree with that. Again, we thank you for the suggestion.

REFEREE: 2. Practitioners always prefer to see the confidence interval. Bartlett corrected confidence interval of the regression coefficients under logistic regression can be found in Das, U., Dhar, S. S., & Pradhan, V. (2018). Corrected likelihood-ratio tests in logistic regression using small-sample data. Communications in Statistics-Theory and Methods, 47(17), 4272-4285. Please use the approach to construct the interval estimate of the parameters.

AUTHORS' REPLY: Thank you. We followed your suggestion and computed confidence intervals for the parameters that index Model $\mathscr{M}_{2R}$ using the following test statistics: ω, ω_{b3} and ω_{a1}. They are reported in Table 8 together with the usual asymptotic confidence intervals for $1-\alpha = 0.95$. We followed Das et al. (2018, Section 3) for obtaining interval estimates based on the test statistics. In particular, for each parameter and each test statistic, we found the set of parameter values such that the test statistic is smaller than $\chi^2_{1;1-\alpha}$. For instance, the confidence intervals for β_5 constructed using ω_{b3} and ω_{a1} are $[-0.0796, -0.0169]$ and $[-0.0781, -0.0145]$, respectively; the corresponding asymptotic interval estimate is $[-0.0749, -0.0216]$. It is noteworthy that none of the reported confidence intervals contains the value zero. We thank you for motivating us to perform this analysis.

REFEREE: 3. You have nuisance parameters in your model. It will be nice to have a discussion in the light of a fundamental work: Ferrari, S. L., Lucambio, F., & Cribari-Neto, F. (2005). Improved profile likelihood inference. Journal of Statistical Planning and Inference, 134(2), 373-391. I know this is a good chunk of work, but should be mentioned about this possibility in your paper.

AUTHORS' REPLY: Thank you. We now cite Ferrari et al. (2005). The authors obtained the Bartlett correction factor in generalized linear models from a likelihood function that is modified to account for the impact of nuisance parameters on the inference made on the parameters of interest. It is a novel approach. We do not pursue it in our manuscript because the derivations become much more cumbersome, especially when the location and precision parameters are not orthogonal, as it is in the case in the class of beta regressions. Additionally, the numerical evidence we report show that our Bartlett corrected test delivers very accurate inferences even when the number of nuisance parameters is large. Nonetheless, we agree with you that it is important to mention the approach

developed by these authors, and we do so in the revised version of the manuscript. We thank you for the suggestion.

REFEREE: 4. Simulation results use predictors generated from independent uniform distribution. In addition to that, i am interested to see the performance of the proposed methods when the predictors will be generated from multivariate normal distribution with different pairwise correlation structures: low correlation (say -/+ 0.1), medium correlation (say -/+ 0.5) and decaying correlation (say $0.25^{|i-j|}$)

AUTHORS' REPLY: Thank you. We followed your suggestion and performed additional simulations in which we varied the degree of correlation between two regressors. We did not consider decaying correlation because the responses are independent and there is no natural ordering for the observations (as it would have been the case with time series or spatially correlated data). We generated the values of the regressors from the bivariate normal distribution with mean vector equal to zero, variances equal to one and correlation (ρ) equal to 0.1, 0.5, 0.75 and 0.95. The correlation strength between covariates thus ranges from very weak to very strong. The results (tests' null rejection rates) are presented in Table 7. They show that the impact of ρ on the tests' small sample behavior is minor. The performances of the Bartlett-corrected are especially insensitive to the strength of the correlation between regressors. Again, ω_{b3} is the best performer. We thank you for motivating us to perform this analysis.

REFEREE: 5. Correct the sentence in lines 186, 207 etc. and read carefully the English

AUTHORS' REPLY: We made the appropriate corrections in the indicated lines. We also made other small corrections that were indicated by Referee 2. Thank you.

We thank Referee 1 for carefully reading our manuscript and providing us with thoughtful comments and suggestions.

REFEREE 2

REFEREE: In this paper, the authors examine beta regression linear models, which include a linear model for the mean term and another linear model for the precision allowing different values for the latter in each observation. Bartlett corrections are used for the inference involving likelihood ratio tests to improve the asymptotic distribution of the statistic considering that small sample sizes are used. Three variants of these corrections are presented and compared through simulation examples. A non-simulated data set concerning intelligence and prevalence of atheism is fitted and tests are applied.

AUTHORS' REPLY: Thank you for accurately summarizing our work.

REFEREE: I enjoyed readying this paper. In general, it is well structured and counts a complete full story. The problems I found are mainly in terms of improving it in how it is read and to interest more the reader in what they are telling us. I found some problems concerning redaction in some paragraphs, as well as in terms of the use of English, particularly in the Introduction, which could be easily corrected by careful readying and editing the complete document. I think that one problem it has now is that it is not easy to find all the main contributions from the beginning, some are hidden in different parts of the paper. Also, there is lack of explanation of where and why certain model aspects are considered, for instance in terms of the link functions used or why having a different precision parameter in each observation is relevant.

AUTHORS' REPLY: Thank you. As you will see below, we took all of your suggestions into account and made changes to the text. The manuscript reads a lot better now. We thank you for carefully reading our paper and providing us with useful feedback.

REFEREE: 1) In line 50 it is stated that varying precision beta regressions and corrections are commonly used. It would be useful for the reader to know in which areas they are used or general examples, including a motivation of why the precision parameter could vary. It seems that this term is somehow similar to heteroscedasticity in linear models, maybe a link with these models could be useful as a motivation. In the example, the readers analyze incidences (rates or risks), thus these beta models can be used for that type of data, but what advantages they have over Poisson models with offset? Or logistic models? (with varying or not precision terms) or in what type of data they are preferred?

AUTHORS' REPLY: Thank for your comments and suggestions. We included references to applied papers that use the beta regression model. We also note in the text that the model is heteroskedastic even in the fixed precision formulation since the variance of y_i changes with μ_i. We note in the revised version of the manuscript that more flexibility can be achieved by allowing the precision parameter to be impacted by some covariate values. In such a more general formulation, the variance of y_i is no longer restricted to be a multiple of $\mu_i(1-\mu_i)$. Thank you for motivating us to comment on that.

REFEREE: 2) In line 60 the example is presented. It could be useful from the beginning specifying what the response is. In general, in the paper the authors present with more detail the explanatory variable (intelligence) than the response. The response is what justifies the use of the specific distribution and this should be presented earlier. Also, I am not sure whether religious belief is an appropriate name for the variable, though I am not expert, perhaps prevalence of atheism as stated in the results is a better name.

AUTHORS' REPLY: Thank you. We followed your suggestion. We now define the dependent (response) variable in the paper Introduction. It reads: "The variable of interest (response) is the proportion of atheists in each country and the covariates include average intelligence and other control variables."

REFEREE: 3) In line 80, the authors talk about the "rate at which size distortions vanish". Later in the paper, they explain what this refers to in lines 135 to 136. I think that it should be explained earlier, considering that as I understand it is one contribution in this paper. In fact, something that it is not clear is whether some of the improved test statistics are proposed by the authors for this paper or not. It seems that the authors calculated all the matricial formulas, which involved a lot of algebra and calculus, for this particular beta model, but not the statistics. The authors should be more emphatic from the introduction about their contribution, in terms of being clear that the derivation and all mathematical calculations for the model are their original work, and if they proposed any statistic for this paper or not, they should be clearer.

AUTHORS' REPLY: Thank you. We followed your suggestion. We now mention in Section 1 (Introduction) that the size distortions of our corrected tests vanish at rate $O(n^{-2})$ whereas those of the alternative tests we consider do so at rate $O(n^{-1})$. We also make clear that our contribution is that we obtain closed-form expressions for the quantities that allow improved testing inferences to be carried out in varying precision beta regressions.

REFEREE: 4) After line 84, the authors introduce the two models and though it can be inferred that they are talking about a beta distribution, I think that in terms of presenting a complete model they should state which distribution is assumed for the response. I understand that the precision parameter does not have an associated distribution, but it could seem as so, when considering how the model is presented. In a similar note, functions g_1 and g_2 are not discussed in practical terms, is it used the canonical link for g_1? Or what functions are used or allow having estimators with better statistical properties? In the simulations, some functions are used and in the example others, and the reader is a little confused of what function should be used.

AUTHORS' REPLY: We now make clear that y_i follows the beta distribution in the first sentence of the section. We also comment on common choices for g_1 and g_2 and provide a reference. Since the beta regression model does not belong to the class of generalized linear models, there is no canonical link.

REFEREE: 5) It seems all notation in the section starting in line 101 is in terms of the beta regression model, which is fine, but the reader is not sure which results are general (which seems to be all page 4) and which ones are particular for these models (which seems to be most of page 5). Maybe if the authors were more explicit saying at the beginning that they are using for simplicity notation concerning the beta models, but that the results apply for any model, and afterwards stating which results are particular for their beta models.

AUTHORS' REPLY: Thank you. We followed your suggestion and tried make clear which results are of general nature and which results relate to the beta regression model.

REFEREE: 6) Notation can become difficult, maybe in some parts it could be useful to remind the reader the meaning of some letters. For instance, after the formula w_{b1} (after line 109), they could say: "whose expected value equals the number of parameters of interest l"

AUTHORS' REPLY: Thank you. We now refer to ω_{b1}, ω_{b2} and ω_{b3} as 'ratio-like', 'exponentially adjusted' and 'multiplicative-like', respectively.

REFEREE: 7) In page 5, after line 116 the first and second sentence seems out of context, it seems that it belongs to a review of the literature. I think the idea here was to introduce that the Bartlett-corrected tests have been obtained in different models, and that now they are obtaining them for beta regressions. All this is fine, but maybe it is more appropriate for the Introduction. In fact, in a sentence there, they clearly state which is their main goal and contribution, which could be stated in the Introduction. Here, they could introduce a sentence saying that from there on what they present is their own work (as I understand all the previous information in this section is known and already used).

AUTHORS' REPLY: Thank you. We followed your suggestion. We removed the remark you mentioned from the text and we also make clear that what follows is our main contribution to the literature on Bartlett corrections.

REFEREE: 8) In lines close to those of the previous comment, the authors state that the beta and delta parameters are not orthogonal, but I was not sure why this state is true.

AUTHORS' REPLY: Thank you. We now explain that β and δ are not orthogonal in the sense that Fisher's information matrix is not block diagonal. We use the definition given in Cox and Reid (1987); see Equation (1) in their paper.

REFEREE: 9) The Bartlett correction test statistics seem variants or functions of the correction c ¿why they were used as such? is one of these corrections proposed specifically for this paper? It seems that they were not.

AUTHORS' REPLY: Thank you. The corrected statistic ω_{b1} is the standard Bartlett-corrected likelihood ratio test statistic. In addition to it, we consider two other Bartlett-corrected test statistics thar are used in Lemonte et al. (2010). In order to use the three test statistics in a given class of models, it is necessary to obtain closed form expressions for ε_k and ε_{k-l} that are valid for such models. For varying precision beta regressions, these quantities can be computed using Equation (4), which is our main result. This is now clear in the revised version of the manuscript. Again, thank you.

REFEREE: 10) For w_{a2} in line 128 it is not explained the meaning or the formula associated with letter ξ.

AUTHORS' REPLY: Thank you for noticing that. ξ is a function of several model-based quantities (score function, expected information, observed information, etc.). Closed-form expressions for ξ were derived by several authors considering different underlying models. This is now explained in the manuscript.

REFEREE: 11) In line 214, it could be specified which values are used. Here they use the same value for β_4 and γ. Would having different values affect the results?

AUTHORS' REPLY: Since these are power simulations (i.e., simulations in which the null hypothesis is false), the true parameter value must be different from the value under test, i.e., data generation has to be carried out using a non-null value for β_4 since we test that β_4 equals zero. This is now better explained in the text, where we now read: "We test $\mathscr{H}_0 : \beta_4 = 0$ (first scenario), but the data are generated using using a value of β_4 that is different from zero; we denote such a value by γ." Thank you for asking us to clarify that.

REFEREE: 12) In line 265 it is stated that only some countries were used for the analysis, but since you are studying Scenario 3, countries with more atheists, I think the results are probably biased to countries that already have a greater development, and care should be taken in some of the conclusions that are provided at the end of the Example.

AUTHORS' REPLY: Thank you. We use 50 countries in our empirical analysis. The sample includes several developing countries, such as Albania, Cambodia, China, Kazakhstan, Mozambique, Russia, Singapore, South Korea, Trinidad and Tobago, Ukraine, Uruguay, Vietnam, and others. Hence, we do not feel that the results are biased to countries that already have a greater development. Additionally, Cribari-Neto and Souza (2013) showed that the results obtained with 50 and 124 countries are similar. We chose to focus on the former because our interest is in small sample inferences and also because by doing so we avoid working with countries with very small prevalence of atheists. We agree, nonetheless, that all empirical results must be accepted and interpreted with care.

REFEREE: 13) In lines 268-275 it is not clear whether these variables are available in the same provided reference.

AUTHORS' REPLY: Thank you. Except for x_8, these are the same variables used by Cribari-Neto and Souza (2013). This is now clearly stated in the text.

REFEREE: 14) After the model in line 275 the authors mention an improved fit, but not what statistic was used.

AUTHORS' REPLY: Thank you. We now note that the fit improvement is verified by three standard model selection criteria and also by the pseudo-R^2. After presenting the model we fit to the data (Model $\mathscr{M}_2$), we comment on the values of three model selection criteria (AIC, AICc and BIC) and of the pseudo-R^2 for each model (Models $\mathscr{M}_1$ and $\mathscr{M}_2$). We thank you for your comment.

REFEREE: 15) Lines 281-282 includes an interaction, but it was not used after that, or perhaps I was not able to understand the redaction of this sentence. In fact, all sentence from line 280 to 282 should be rewritten. I think the authors wanted to say here that they tested whether β_4 and β_5 were zero in the model.

AUTHORS' REPLY: Thank you. The two interaction variables (x_7 and x_8) are used as mean regressors in our model and one of then (x_7) is a mean regressor in the model used by Cribari-Neto and Souza (2013). As for your second remark, in the revised version of the manuscript we make clear that we wish to test whether β_4 and β_5 equal zero in the model. Again, thank you.

REFEREE: 16) Maybe to improve the redaction from line 317, the models can be rewritten using the notation M_1 for the model fitted in reference [7] and M_2 for the model proposed by the authors.

AUTHORS' REPLY: Thank you. We followed your suggestion. We now refer to the model used by Cribari-Neto and Souza (2013) as Model $\mathscr{M}_1$ and to the models we consider in our analysis as Models $\mathscr{M}_2$ and $\mathscr{M}_{2R}$ (reduced version). Again, we thank you for the suggestion.

REFEREE: 17) As I understand, and from a point of view of medical studies, intelligence is your explanatory variable and the others are variables you want to control for, is that so? If it is, maybe it could be said.

AUTHORS' REPLY: Thank you. We followed your suggestion. It is now stated in the text that average intelligence is the independent variable of main interest and the remaining regressors are control variables.

REFEREE: 18) Was possible multicollinearity analyzed in the example? This is an important feature that should be analyzed.

AUTHORS' REPLY: We use the same regressors as Cribari-Neto and Souza (2013). Unlike what happens in linear regressions, multicollinearity is not a severe problem in beta regressions since neither the parameter vector estimator not its covariance matrix requires inverting $X'X$, where X is the matrix of regressors. This can be seen, for example, in the set of numerical results we included in the manuscript in response to a suggestion made by Referee 1. We now report simulation results obtained using different levels of correlation between two mean regressors. As you can see, the inferential results (Table 7) are quite robust to strong correlation between regressors ($\rho = 0.95$). The performances of the Bartlett-corrected are especially insensitive to the strength of the correlation between regressors. Again, ω_{b3} is the best performer.

REFEREE: 19) Lines 352-354 point out a contribution, in terms of these particular data, but still a contribution. I am not sure whether this could added to the discussion or somewhere more visible.

AUTHORS' REPLY: Thank you. We followed your suggestion and we now mention the contribution of our empirical analysis to the literature in Section 1 (Introduction). Thank you for motivating us to do that.

REFEREE: 20) I think it could be interesting to make available, perhaps as supplementary material, the R code related to the simulations or the real data example. And by the way, is the data set fully available?

AUTHORS' REPLY: Thank you. As per your suggestion, we are now making the data and the computer code used in our empirical analysis publicly available. They can be obtained from `https://github.com/acguedes/beta-Bartlett`. This is information is in the revised version of our manuscript. Again, thank you.

REFEREE: 21) Something I was wondering is what happens when the response variable is heavily clustered around or is zero? Can the models still be used or modified? Can your results be generalized for any member of the exponential family? If the answer involves a new research question, it could be relevant to include in the discussion some of these aspects or others as future work and perspectives.

AUTHORS' REPLY: The beta regression model is useful for modeling responses that assume values in $(0,1)$. Hence, response values that are exactly equal to zero or one are not allowed. When such values are present in the data, it is necessary to use a regression model that combines continuous and discrete components such as the inflated beta regression model introduced by Ospina and Ferrari (2012). Neither model (beta and inflated beta) is a generalized linear model. Following your suggestion we included a direction for future research in the concluding remarks section: "A direction for future research is the extension of our analytical results for testing inferences in inflated beta regression models introduced by Ospina and Ferrari (2012) which include both continuous and discrete components and thus allow for response values that are exactly equal to 0 or 1."

REFEREE: 22) Authors should check that reference numbers, particularly for sections and subsections is correct, and that they appear in the document.

AUTHORS' REPLY: Thank you. The numbers of the of references were automatically generated by BibTeX. We double checked all references, though. Thank you for suggesting that.

REFEREE: Line 4, it seems it should say "of interest"

AUTHORS' REPLY: We made the correction. Thank you.

REFEREE: Line 9, perhaps it should be "Its density function"

AUTHORS' REPLY: You are, of course, correct. We made the correction. Thank you.

REFEREE: Line 12 , which authors?

AUTHORS' REPLY: We changed the text to make it clear that we are referring to [1]. Thank you.

REFEREE: Line 16, "set of"

AUTHORS' REPLY: We made the correction. Thank you.

REFEREE: Line 107 There is something odd in how all the symbols and redaction are presented.

AUTHORS' REPLY: We rewrote the sentence. We hope it is clearer now. Thank you.

REFEREE: After line 109, there is something strange in presenting just the number to start the paragraph, maybe something like "In [11] it was proposed"

AUTHORS' REPLY: We changed the text according to your suggestion. Thank you.

REFEREE: After line 120 tr() should be used instead of tr{}

AUTHORS' REPLY: We made the suggested correction. Thank you.

REFEREE: Line 162. Perhaps it should say "ratio test ω"

AUTHORS' REPLY: Thank you. We followed your suggestion.

REFEREE: Lines 186-187 have several redaction problems.

AUTHORS' REPLY: We changed the text. Thank you.

REFEREE: Line 200. Perhaps "consider" instead of "move to"

AUTHORS' REPLY: We followed your suggestion and changed the text accordingly. Thank you.

REFEREE: Line 202. Perhaps could be helpful to remind the reader of the meaning of fixed precision, for instance adding "that is, we test whether the precision is a constant"

AUTHORS' REPLY: We followed your suggestion. The text now reads: "We test the null hypothesis of fixed precision, i.e., we test $\mathscr{H}_0 : \delta_2 = \delta_3 = 0$ which is equivalent to testing $\mathscr{H}_0 : \phi_1 = \cdots = \phi_n = \phi$ (the precision parameter is constant across observations)." Thank you.

REFEREE: Line 336. All redaction was strange here, I think they wanted to say that “In [7], Figure 4, the author plotted”, meaning that the authors in that reference have a Figure 4.

AUTHORS’ REPLY: Thank you. We changed the text according to your suggestion.

We thank Referee 2 for carefully reading our manuscript and providing us with thoughtful comments and suggestions.

References

D. R. Cox and N. Reid. Parameter orthogonality and approximate conditional inference. *Journal of the Royal Statistical Society B*, 49(1):1–39, 1987.

F. Cribari-Neto and T. C. Souza. Religious belief and intelligence: Worldwide evidence. *Intelligence*, 41:482–489, 2013.

U. Das, S. S. Dhar, and V. Pradhan. Corrected likelihood-ratio tests in logistic regression using small-sample data. *Communications in Statistics - Theory and Methods*, 47(17):4272–4285, 2018. doi: 10.1080/03610926.2017.1373815.

S. L. P. Ferrari, F. Lucambio, and F. Cribari-Neto. Improved profile likelihood inference. *Journal of Statistical Planning and Inference*, 134(2):373–391, 2005. doi: 10.1016/j.jspi.2004.05.001.

A. J. Lemonte, S. L. P. Ferrari, and F. Cribari-Neto. Improved likelihood inference in Birnbaum-Saunders regressions. *Computational Statistics & Data Analysis*, 54(5):1307–1316, 2010.

R. Ospina and S. L. P. Ferrari. A general zero-or-one inflated beta regression models. *Computational Statistics & Data Analysis*, 56(6):1609–1623, 2012. doi: 10.1016/j.csda.2011.10.005.
